# The Biology and Targeting of FLT3 in Pediatric Leukemia

**DOI:** 10.3389/fonc.2014.00263

**Published:** 2014-09-23

**Authors:** Colleen E. Annesley, Patrick Brown

**Affiliations:** ^1^Oncology and Pediatrics, The Sidney Kimmel Comprehensive Cancer Center, Johns Hopkins University School of Medicine, Baltimore, MD, USA

**Keywords:** FLT3, FLT3 inhibitor, MLLr, MLL, tyrosine kinase inhibitors

## Abstract

Despite remarkable improvement in treatment outcomes in pediatric leukemia over the past several decades, the prognosis for high-risk groups of acute myeloid leukemia (AML) and acute lymphoblastic leukemia (ALL), as well as for relapsed leukemia, remains poor. Intensification of chemotherapy regimens for those at highest risk has improved success rates, but at the cost of significantly increased morbidity and long-term adverse effects. With the success of imatinib in Philadelphia-chromosome-positive leukemia and all-*trans* retinoic acid in acute promyelocytic leukemia, the quest to find additional molecularly targeted therapies has generated much excitement over recent years. Another such possible target in pediatric acute leukemia is FMS-like tyrosine kinase 3 (FLT3). FLT3 aberrations are among the most frequently identified transforming events in AML, and have significant clinical implications in both high-risk pediatric AML and in certain high-risk groups of pediatric ALL. Therefore, the successful targeting of FLT3 has tremendous potential to improve outcomes in these subsets of patients. This article will give an overview of the molecular function and signaling of the FLT3 receptor, as well as its pathogenic role in leukemia. We review the discovery of targeting FLT3, discuss currently available FLT3 inhibitors in pediatric leukemia and results of clinical trials to date, and finally, consider the future promise and challenges of FLT3 inhibitor therapy.

## Introduction

Remarkable strides have been made in the past several decades in the treatment success rates of childhood acute lymphoblastic leukemia (ALL), with 5-year survival rates now approaching 90% (1). However, up to 20% of children will be refractory to treatment, or relapse following treatment, and the event-free survival (EFS) rate for these patients remains poor. In acute myeloid leukemia (AML), although the majority of children will achieve an initial remission with conventional chemotherapy, <60% will be long-term survivors (2). Furthermore, the intensification of treatment regimens required to achieve the highest possible survival rates for poor risk leukemia has been pushed to the limit of tolerance, highlighting the need for effective targeted therapies (3).

Two successful examples of targeted therapies in pediatric leukemia include tyrosine kinase inhibitors in BCR-ABL-positive ALL (4) and all-*trans* retinoic acid (ATRA) in acute promyelocytic leukemia (APML) with the PML–RARα fusion (5). FMS-like tyrosine kinase 3 (FLT3) represents another attractive target, given its overexpression on the majority of leukemia cells and the high rate of FLT3 mutations in human leukemia. Since the receptor was first described over 20 years ago, targeting FLT3 therapeutically has generated much excitement. The first clinical trials with FLT3 inhibitors took place 10 years ago, and though some inhibitors have shown good promise in effective targeting, they also presented several clinical challenges. This is underscored by the fact that no FLT3 inhibitors have been FDA-approved for the treatment of leukemia to date. This review will summarize the biology of FLT3 in leukemia, and discuss the benefits and hindrances associated with FLT3 inhibitor therapy.

## Biology of the FLT3 Receptor

### Molecular structure and normal tissue expression of FLT3

**Figure 1:**
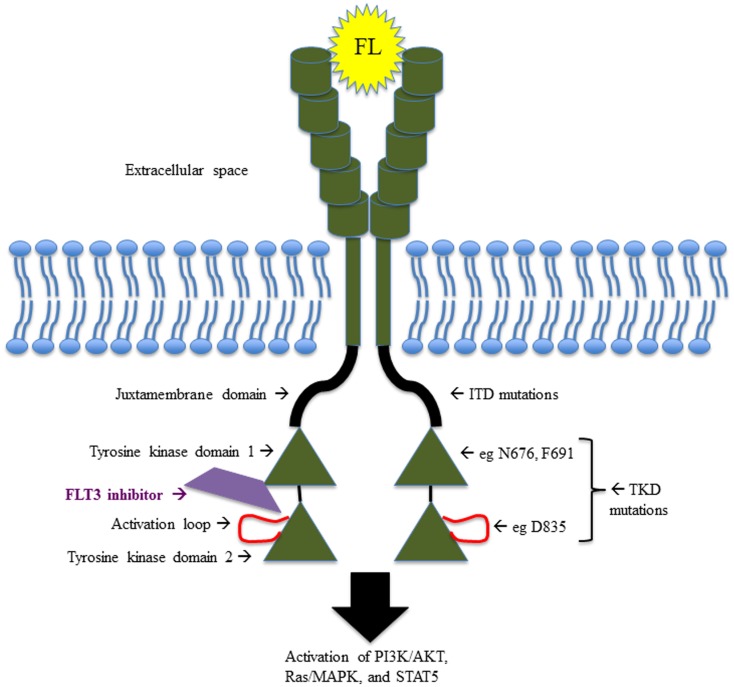
**Schematic illustrating the structure and function of FLT3, including the sites of the most common activating mutations**.

FMS-like tyrosine kinase 3 belongs to the class III receptor tyrosine kinase (RTK) family, along with KIT, FMS, and platelet-derived growth factor receptor (PDGFR). As such, FLT3 contains an extracellular domain made up of five immunoglobulin-like regions at the amino terminus, a single transmembrane region, an intracellular juxtamembrane domain (JMD), and two kinase domains at the carboxyl terminus, separated by a kinase insert region (6, 7) (Figure 1). FLT3 is expressed in normal human bone marrow (BM), particularly in CD34^+^ hematopoietic stem and early progenitor cells (6, 8) and in dendritic cell progenitors (9). FLT3 is also expressed in human brain, placenta, and testis (7, 10), though its function in these tissues remains unclear.

### Normal receptor function

FMS-like tyrosine kinase 3 signaling is central to the development of hematopoietic stem cells, B-cell progenitors, dendritic cell progenitors, and natural killer (NK) cells. This was first demonstrated through studying the targeted disruption of either FLT3 or its ligand, FLT3 ligand (FL), in CD34^+^ cells or in mice (8, 11, 12). Mice homozygous for a deletion of FLT3 mature into normal adults, but BM evaluation reveals deficiencies in B-cell progenitors, and transplantation studies show deficiencies in T-lymphocytes and myeloid cells (11). In colony-forming assays, human CD34^+^FLT3^high^ BM cells give rise to colony-forming unit granulocyte-monocyte (CFU-GM) colonies and are predominantly in G1 phase of cell cycle, whereas CD34^+^FLT3^low^ cells give rise to erythroid colonies and are predominantly in G0 phase (13). Together, these data reveal the significant role of FLT3 in both differentiation and proliferation of hematopoietic progenitor cells.

#### FLT3 ligand

FMS-like tyrosine kinase 3 ligand was described shortly after the discovery of the FLT3 receptor (14, 15). FL is expressed in many different human tissues, though its co-expression with FLT3 is limited to the gonads and hematopoietic tissue (16). FL is also produced by BM stroma, an important source of cytokines and growth factors responsible for the proliferation and differentiation of hematopoietic progenitor cells (17). It is found in both soluble and membrane-bound forms, and selectively stimulates the proliferation and colony formation of CD34^+^ progenitor cells (16). Upon binding to FLT3, FL induces dimerization of the receptor and auto-phosphorylation of tyrosine residues in the kinase domains, leading to downstream activation and phosphorylation of protein substrates (18).

#### FLT3 signaling pathways

Upon stimulation with FL, FLT3 activation results in the downstream activation of multiple signaling pathways, including the Ras/Raf and the phosphatidylinositol 3′ kinase (PI3K) pathways. Many important signaling and adaptor proteins are involved, including signal transducer and activator of transcription 5 (STAT5), phospholipase Cγ (PLC-γ), CBL, growth factor receptor-bound protein 2 (Grb2), SHC, Src-homology 2 containing protein tyrosine phosphate (SHP-2), Src-homology 2 containing inositol phosphatase (SHIP), mitogen activated protein kinase (MAPK), and extracellular-signal regulated kinase (ERK1/2) (19–24). This cascade of phosphorylation and activation ultimately results in increased cell proliferation, decreased cell apoptosis, and inhibition of cell differentiation.

## FLT3 Aberrancies in Leukemia

FMS-like tyrosine kinase 3 is aberrantly expressed in all precursor B-cell (pre-B) ALL and nearly all AML primary leukemia samples, as well as a fraction of T-cell ALL patient samples (25, 26). FLT3 is also expressed in the majority of pre-B ALL and AML cell lines (27–29). FLT3 is expressed in most chronic myeloid leukemia (CML) blast crisis patient samples, irrespective of phenotype, as well as in several chronic lymphocytic leukemia (CLL) samples (26). Importantly, FLT3 is expressed on leukemia blasts regardless of CD34 expression, whereas FLT3 expression is limited to the CD34^+^ population in normal human BM (26, 27). This pattern of FLT3 expression in leukemia is in contrast to KIT and FMS expression, which are typically restricted to myeloid leukemia (25). FL is expressed in most leukemia cell lines from all cell lineages, and in one study, 40 of 110 human leukemia cell lines expressed both FL and FLT3 (27). Another study showed that wild-type FLT3 is constitutively activated in two-thirds of AML patient samples and in 4 of 13 leukemia cell lines tested (30). These cells co-expressed FLT3 and FL, suggesting a role for autocrine signaling.

### FLT3-activating mutations

Activating mutations of FLT3 were first described in AML in 1996. There are two broad categories: internal tandem duplications (ITD) and point mutations in the tyrosine kinase domain (TKD). In pediatric AML, activating mutations occur in 20–25% of patients, with roughly two-thirds of these harboring ITD mutations, and the remaining one-third with TKD point mutations.

#### ITD mutations

Internal tandem duplications mutations in the JMD of the FLT3 gene were first described in adult patients with AML, and even in that small cohort of patients, it appeared that these mutations conferred a poor prognosis (31). A region of the JMD coding sequence is replicated in a direct head-to-tail orientation, creating the ITD. As initially described, ITDs vary in length and location, but tend to occur in replicates of three nucleotides, maintaining an intact reading frame and not affecting other regions of the gene, including the kinase domains (31–33). ITD mutations result in ligand-independent dimerization of the FLT3 receptor, constitutive phosphorylation, and activation of the kinase domains (32, 34, 35). It is proposed that ITD mutations occur as DNA replication errors, and are selected due to the resulting growth advantage (32).

In both AML and normal hematopoiesis, self-renewal capacity is limited to the immature CD34^+^ CD38^−^ cell population (36). There is increasing evidence that FLT3-ITD has activity in hematopoietic stem cells (37), as demonstrated by the presence of ITD in the CD34^+^CD38^−^ leukemia-initiating cell (LIC) population, and confirmation of the ITD mutation in these successfully engrafted LICs in a murine transplant model (38). In BM cells from a knock-in heterozygous FLT3-ITD-mutant mouse model, FLT3 expression and signaling was present within the long-term hematopoietic stem cell (LT-HSC) compartment, and importantly, the ITD mutation conferred a proliferative effect on this normally quiescent population, depleting the LT-HSC compartment (39). Interestingly, this effect was reversed with the FLT3 inhibitor sorafenib, repleting the LT-HSC population and returning these cells to a quiescent state.

##### Clinical relevance of ITD mutations in human leukemia

Internal tandem duplications mutations are a common somatic mutation in adult AML, occurring in approximately 20–35% of patients (31, 40–43). ITD mutations occur less frequently in pediatric AML, found in approximately 5–15% of newly diagnosed patients (44–46). The presence of an FLT3-ITD mutation in pediatric AML is associated with higher WBC counts at presentation, older age, higher rates of induction failure, and significantly worse survival. In a retrospective study of *de novo* AML pediatric patients enrolled in Children’s Cancer Group study (CCG)-2891, multivariate analysis identified ITD mutations as an independent prognostic factor for poor outcome in pediatric AML. This study revealed 8-year overall survival (OS) and EFS rates of 13 and 7%, respectively, for children with ITD mutations versus OS of 50% and EFS of 44% for children without ITD mutations (46). Following these results, the risk stratification of pediatric patients with ITD mutations has been a dynamic process. One pediatric study showed that the presence of FLT3-ITD mutations in the more primitive CD34^+^ CD33^−^ cell population was heterogeneous (present in approximately 80% of ITD-positive AML patients), and conferred a worse prognosis compared to patients in which the ITD mutations was only present in CD34^+^ CD33^+^ cells (47).

Furthermore, in a large retrospective study of adults with AML characterizing patients with FLT3 mutations, adults with higher ITD allelic ratios had significantly shorter overall and disease-free survival (42). A subsequent retrospective study of children with *de novo* AML, enrolled on studies CCG-2941 and -2961, determined that an ITD allelic ratio of 0.4 or higher identified the highest risk group with the worse prognosis, whereas children with allelic ratios <0.4 had similar outcomes as children with wild-type FLT3 (48). Therefore, the current Children’s Oncology Group (COG) phase 3 clinical trial for AML risk-stratifies patients with FLT3-ITD allelic ratios >0.4 into the high-risk group, regardless of having any other low risk factor. These patients will receive more intensive chemotherapy, including the FLT3 inhibitor sorafenib (further discussed below), as well as hematopoietic stem cell transplantation (HSCT) in first remission, in an attempt to improve outcomes for this subset of patients.

Additionally, loss of the FLT3-wild-type allele and resultant copy number-neutral ITD homozygosity has been demonstrated in AML, particularly at disease progression, and is associated with a worse prognosis (49–51). Copy-neutral loss of heterozygosity (CN-LOH) is a common event in both solid and hematologic malignancies, and can lead to either inactivation of a tumor suppressor gene or enhanced activity of an oncogene. Acquired CN-LOH or uniparental disomy, is seen in leukemia with loss of the normal allele and duplication of the mutant allele (52–55), such as seen with FLT3-ITD mutations.

Although ITD mutations were first described in the JMD, it was later discovered that ITD mutations occur up to 30% of the time in non-JMD regions of the gene (56, 57). In fact, in a large review of 241 FLT3-ITD AML patients, those with an ITD in the beta-1 sheet of the TKD had a significantly inferior remission rate, relapse free survival, and OS compared to those with an ITD located in the JMD (57). This suggests relevance to identifying not only the presence and allelic burden of FLT3-ITD but also the ITD location.

There are conflicting reports as to whether the length of the ITD carries prognostic significance in AML. In one retrospective review of adult data, patients harboring an ITD >40 base pairs had an inferior 5-year OS of 13% compared to 26% in those with an ITD <40 base pairs, and 21% in patient with no ITD mutation (58). This group hypothesized that longer ITDs may lead to increased disruption of the auto-inhibitory function of the JMD. However, another group demonstrated opposing results, showing inferior survival in patients with shorter ITDs, using a cutoff of 70 base pairs (59). Prospective, larger studies are likely needed to determine whether ITD size truly affects prognosis.

Finally, a recent report evaluated the prevalence and prognostic significance of FLT3 mutations in childhood APML. It is well known that FLT3 mutations are common in APML, and the block in differentiation conferred by t(15;17) likely cooperates with the proliferative advantage of FLT3 mutations. In 104 pediatric APML patients, 40% had either FLT3-ITD or a TKD point mutation. These patients had higher WBC counts at diagnosis and increased rates of induction deaths when treated with ATRA alone (60). No difference in outcomes with FLT3 mutations were demonstrated post-induction therapy. It is unclear if the increased induction deaths were due to the higher WBC count or FLT3 mutations; however, it is hypothesized that the presence of a FLT3 mutation is causative of the higher WBC counts in these patients. Gene-expression profiling in FLT3-mutant APML showed up-regulation of genes involved in proliferation, coagulation, and inflammation (61). This suggests a role for evaluating FLT3 mutation status in APML and consideration of early FLT3 inhibitor therapy in FLT3-mutant APML patients.

##### Mechanism of ITD mutation-induced leukemogenesis and model systems

Multiple signaling pathways are implicated in aberrant FLT3 activation in cells. The JMD of FLT3 contains auto-inhibitory function and maintains the kinase in an inactive conformation (62). The disruption of this region by an ITD results in constitutive activation of the FLT3 receptor (63). Activation of FLT3 results in the phosphorylation of proteins such as Gab2, SHP-2, and CBL, forming a complex that interacts with the p85 subunit of PI3K, thereby activating the anti-apoptotic PI3K pathway (20, 23). Serine/threonine protein kinase B (AKT) is shown to be phosphorylated and constitutively activated in 32D cells, which have been stably transfected with ITD (64).

The proliferative Ras/MAPK pathway is also activated with FLT3 signaling. Ba/F3 cells transfected with wild-type human FLT3 and stimulated with FL demonstrate transient activation of MAPK and phosphorylation of ERK1/2 (21). MEK inhibition reverses this activation and also inhibits the proliferative effect of FL on these cells. Both 32D and Ba/F3 cells transfected with FLT3-ITD alone show activation of MAPK (64, 65). Finally, MAPK is activated in primary AML blasts (65, 66).

STAT5 is involved in the regulation of self-renewal and differentiation of hematopoietic progenitor cells (67), and its aberrant activation has been demonstrated in human leukemia (68). STAT5 is constitutively phosphorylated and activated in cell lines transfected with FLT3-ITD mutations, as well as leukemia cell lines and primary AML blasts containing endogenous ITD mutations, but STAT5 is not activated in cells with wild-type FLT3, despite stimulation with FL (64, 65, 69, 70). FLT3-ITD-induced STAT5 activation was found to be independent of Src or Jak kinases in cell lines deficient for these kinases, and *in vitro* assays revealed that STAT5 is a direct target of FLT3 (71).

Yet, another potential mechanism of ITD mutations in leukemogenesis is the decreased expression of SHP-1, a phosphatase responsible for the down-regulation of multiple growth factor and cytokine signaling pathways. TF1 cells transfected with ITD induced a threefold decrease in SHP-1 activity (72). Decreased expression of SHP-1 results in increased proliferation in these cells. Also, *in vitro* treatment of FLT3-ITD cell lines and primary AML patient samples with an FLT3 inhibitor resulted in up-regulation of SHP-1 expression. Taken together, these data implicate SHP-1 as a tumor suppressor that has significantly decreased activity in ITD-positive AML (72).

Finally, FLT3-ITD also contributes to a block in cell differentiation. 32D cells transfected with ITD do not undergo granulocyte differentiation in response to granulocyte colony stimulating-factor (G-CSF) (73). Subsequent investigation showed that the expression levels of C/EBPα and PU.1, both important modulators of myeloid differentiation, are repressed in the presence of FLT3-ITD (74). Treatment of these cells with an FLT3 inhibitor restored expression of these proteins and resulted in myeloid differentiation.

While FLT3-ITD has certainly been demonstrated as a transforming lesion in multiple cell models and clearly contributes strongly to leukemogenesis, it is not sufficient to fully transform primary hematopoietic cells or generate leukemia in mouse models. FLT3-ITD expression confers cytokine-independent growth in 32D and Ba/F3 cell lines (64, 65), and induction of the signaling pathways discussed above. Mice injected with these transformed cell lines rapidly developed a leukemia-like illness (64, 66), but transplantation of primary BM cells retrovirally transduced with FLT3-ITD causes only a myeloproliferative disease (MPD) and not leukemia (75). Knock-in of heterozygous mutant ITD into murine FLT3 (FLT3^+/ITD^) causes a fatal MPD characterized by splenomegaly, leukocytosis, and myeloid expansion, but not frank leukemia (76). Consistent with cell models, these mice demonstrate expansion of dendritic cells and a block in B-cell development, caused by inefficient double strand break repair by non-homologous end joining and failure of proper VDJ rearrangement in pro-B cells (77).

The heterozygous FLT3-ITD mutation does, however, cooperate with other molecular lesions in mouse models to generate both myeloid and lymphoid leukemia, such as MLL-AF9 (78), mutant C/EBPα (79), NUP98–HOXD13D (NHD13) fusion (80), and mutant nucleophosmin (NPMc+) (81), in agreement with accumulating evidence that leukemogenesis is a process that requires multiple genetic or epigenetic “hits.” Interestingly, a mouse model of homozygous FLT3-ITD mirrored the increased disease severity of CN-LOH seen in human malignancy with some homozygous mice developing spontaneous leukemia (77). Another group found similar results with homozygous FLT3-ITD knock-in mice, and by crossing these mice with FL knockout mice, also demonstrated that the homozygous FLT3-ITD phenotype is FL-independent, in contrast to the heterozygous FLT3-ITD mice, which had a milder phenotype in the absence of FL (82). These data suggest that ITD gene “dosage” is probably most important in LOH, but that loss of the wild-type allele also plays a functional role. This is in agreement with *in vitro* experiments showing enhanced FLT3 signaling in response to FL in FLT3-ITD-expressing cell lines, but no consistent effects of FL on STAT5 phosphorylation or other signaling pathways in primary AML blasts homozygous for ITD (83).

#### Tyrosine kinase domain point mutations

Point mutations in the activation loop of the TKD of FLT3 were first described in *de novo* human leukemia in 2001 (84, 85). This area of the gene was interrogated for mutations because the corresponding domain in KIT was reported to harbor oncogenic activating point mutations (86). The first TKD mutations described involved aspartic acid residue D835, which is equivalent to D816 mutations in KIT, and less commonly, isoleucine I836. The D835 mutation is a missense mutation and confers a change in amino acid to tyrosine, histidine, valine, glutamic acid, or asparagine. In these newly diagnosed adult AML patients, TKD mutations occurred independently of ITD mutations (85).

##### Clinical relevance of TKD mutations in human leukemia

Tyrosine kinase domain mutations are less common than ITD mutations and do not appear to carry the same prognostic significance in leukemia. In fact, TKD and ITD mutations demonstrated differing gene-expression profiles in newly diagnosed pediatric AML patients (87). In large retrospective studies, TKD mutations were present in ~7% of both adult and pediatric *de novo* AML (42, 48, 85). In contrast to patients with ITD mutations, patients with D835 TKD mutations did not have elevated WBC counts compared to those with wild-type FLT3 (48, 85, 88). In a large adult study, 17 of 979 (1.7%) AML patients were positive for both ITD and TKD mutations, and 4 of 10 of these harbored the mutations on the same allele (42). In one pediatric study, 35% of patients with TKD-mutant AML also harbored an 11q23/MLL rearrangement (MLL-r) (26% had MLL-AF9) in comparison to 24% of FLT3-wild-type and only 2% of ITD-mutant AML patients with MLL-r (48).

Interestingly, in pediatric pre-B ALL, FLT3-TKD mutations are not infrequently associated with MLL-r. In 30 MLL patient samples tested, 5 (16%) had mutations in the activation loop of TKD, and the residues involved were the same as demonstrated in AML, e.g., D835 (89). This group demonstrated that these TKD mutations resulted in constitutive activation of FLT3, an effect that could be reversed with a FLT3 small-molecule inhibitor. This was the first suggestion of clinically relevant TKD mutations in ALL, and implied a role for FLT3 inhibition in this subset of MLL-r patients. FLT3 mutations were also detected in 20–25% of hyperdiploid (>50 chromosomes or DNA index >1.14) diagnostic pre-B ALL samples, and were associated with high levels of FLT3 expression (90, 91). In these small cohorts of hyperdiploid patients, it is unclear if FLT3 mutations affect prognosis, and larger prospective studies would need to determine this. These data nevertheless support a potential role for FLT3 inhibitors in this additional cohort of patients.

Arguably, the most clinically relevant role of FLT3-TKD mutations in leukemia is the development of a secondary point mutation detected at relapse, as a mechanism of resistance in patients with a pre-existing ITD mutation and after FLT3 inhibitor therapy. This important issue is discussed later in this review.

##### Mechanism of TKD mutation-induced leukemogenesis and model systems

In wild-type FLT3, the activation loop of the TKD normally maintains a “closed” conformation, preventing ATP and proteins from binding (63). The presence of a TKD mutation in this area is thought to open the activation loop, allowing for constitutive activation of the receptor. Phenylalanine substitutions for tyrosine residues in the kinase domain in a TKD-mutant mouse model revealed three critical residues involved in stabilizing the active conformation of the activation loop, one of which is the site of auto-phosphorylation in TKD mutants (92). Functional studies in Cos7 and 32D cells demonstrate that D835 TKD mutations result in auto-phosphorylation and constitutive activation of FLT3 independent of FL, in a similar manner to ITD mutations. D835 mutations also confer cytokine independence in 32D cells (85), further evidence of their gain of function, and transforming capability. Yamaguchi et al. also reported a robust increase in IL-3-independent proliferation in 32D cells expressing both TKD mutations and MLL-AF4 (93).

A murine BM transplant model using retroviral transduction compared disease phenotypes of FLT3-ITD and FLT3-TKD mutations *in vivo*, and surprisingly, TKD mutations caused an oligoclonal lymphoid disorder (both B- and T-lymphoid) compared to MPD seen with ITD mutations (94). The disease latency was longer than with ITD mutations, and mice with TKD mutations did not develop leukocytosis. Some myeloid expansion was seen in TKD-mutant BM, although the primary disease phenotype was lymphoid. In transfected Ba/F3 and 32D cells, AKT and ERK1/2 were similarly phosphorylated with ITD and TKD mutations, but STAT5 was significantly more phosphorylated with the ITD mutation in both cell lines, and in transduced BM cells (94). In a recent report, a knock-in mouse model heterozygous for the D835Y mutation was generated, and shown to survive significantly longer than heterozygous FLT3-ITD knock-in mice (95). In contrast to the first model, in which FLT3 retroviral transduction likely resulted in overexpression of FLT3, the majority of these knock-in mice did develop myeloproliferative neoplasms (MPN), albeit with a less aggressive phenotype than the FLT3-ITD mice. Also, whereas a significant block in B-cell development and drastic reduction in the LT-HSC compartment were demonstrated in the FLT3-ITD mice, neither was observed in the FLT3-D835Y mice. Consistent with the other mouse model, STAT5 was significantly more phosphorylated in FLT3-ITD mice compared to FLT3-D835Y mice (95), suggesting that differential STAT5 signaling may explain the different phenotypes seen between FLT3-TKD and FLT3-ITD leukemia.

### FLT3 overexpression in leukemia

Gene-expression profiling of patients samples comparing pre-B ALL with MLL-r, pre-B ALL without MLL-r, and AML demonstrated that MLL-r leukemia contains a unique signature (96). MLL-r leukemia samples generally expressed high levels of genes associated with hematopoietic progenitor cells, suggesting that MLL is arrested at an earlier stage of development. Armstrong et al. found that the most consistently and impressively overexpressed gene in MLL-r leukemia is FLT3.

#### MLL-r infant ALL

Expanding on their gene-expression profiling data, Armstrong et al. found that overexpression of wild-type FLT3 is an activating lesion in SEMK2, a t(4;11) MLL-r pre-B ALL cell line (89). Strikingly, FLT3 is basally phosphorylated and constitutively activated in this cell line, to an even higher extent than in the AML cell line MV4;11, which contains an FLT3-ITD mutation as well as t(4;11). This was in contrast to two pre-B ALL cell lines without MLL-r or FLT3 overexpression, and the pre-B ALL cell line RS4;11 which contains t(4;11) but does not demonstrate FLT3 overexpression. FLT3 overexpression in SEMK2 cells is due to intra-chromosomal amplification of the gene locus, with each cell containing one to two amplicons of 10 copies of the gene. An FLT3 small-molecule inhibitor demonstrated selective cytotoxicity and reversal of FLT3 activation in SEMK2 and MV4;11 cells, and showed anti-tumor effect *in vivo* in a leukemia mouse model engrafted with SEMK2, but not with RS4;11 cells (89). It was then shown that CEP-701 (lestaurtinib), a small-molecule FLT3 inhibitor, is selectively cytotoxic in infant ALL patient samples with MLL-r and FLT3 overexpression, as well as in FLT3-overexpressing hyperdiploid ALL (97, 98). Lestaurtinib has also been shown to reverse FLT3 auto-phosphorylation in FLT3-overexpressing ALL patient samples (97). Importantly, subsequent studies of infant ALL patients revealed a worse overall prognosis with high levels of FLT3 expression in MLL-r leukemia (99, 100). These data serve as the pre-clinical rationale for FLT3 inhibition in FLT3-overexpressing MLL-r ALL in infant leukemia.

#### Hyperdiploid ALL

Gene-expression analysis of a large number of diagnostic pediatric ALL cases also demonstrated overexpression of FLT3 in hyperdiploid (>50 chromosomes) samples (101). To our knowledge, there is no pre-clinical or clinical study of FLT3 inhibition in hyperdiploid ALL, possibly due to the relatively good clinical outcomes of these patients with traditional chemotherapy.

#### Mechanism of FLT3 overexpression-induced leukemogenesis and model systems

Constitutive FLT3 signaling and the leukemogenic role of FLT3 overexpression in these subsets of ALL is likely caused either by concomitant FLT3-TKD mutations, or the co-expression and autocrine signaling of FL. In model systems, Ba/F3 cells overexpressing wild-type FLT3 did not become cytokine-independent, in contrast to FLT3-mutant-expressing cells. However, FLT3 overexpressing Ba/F3 cells engrafted into Balb/c mice did cause leukemia, which may have been secondary to murine FL expression in this model (69). The transplantation of Balb/c mice with FL-expressing BM also caused leukemia, albeit with a longer latency (102), again suggesting an autocrine or paracrine role of FL in these models.

## FLT3-Targeted Therapy

### Discovery, pre-clinical evidence and pharmacodynamics assessment of FLT3 inhibitors

#### First small-molecule inhibitors: *in vitro* proof of principle

Similar to the small-molecule tyrosine kinase inhibitor imatinib, FLT3 inhibitors act by competitively binding to the ATP-binding site. The first FLT3 small-molecule kinase inhibitors reported were AG1295 and AG1296, which were selectively cytotoxic to cell lines transfected with FLT3-ITD and primary patient samples with ITD mutations, and demonstrated inhibition of downstream FLT3 signaling *in vitro* (103, 104). Despite achievable IC50’s in the nanomolar range, these initial inhibitors are not soluble and therefore not biologically available.

CEP-701 (lestaurtinib) is an orally available indolocarbazole derivative with activity against multiple kinase receptors. It was discovered through a small-molecule inhibitor library screen, and was subsequently found to selectively inhibit auto-phosphorylation of constitutively active FLT3 *in vitro*, as well as prolong survival of an FLT3-ITD mouse model *in vivo* (66). Furthermore, lestaurtinib was selectively cytotoxic to FLT3-ITD primary pediatric AML samples compared to FLT3-wild-type or FLT3-TKD-mutant patient samples (105). Aberrant overexpression of wild-type FLT3 in infant MLL-r and hyperdiploid ALL can cause constitutive FLT3 activation; therefore, lestaurtinib was next investigated in cell lines and primary patient samples with these lesions. Selective cytotoxicity, inhibition of FLT3 phosphorylation, and overall suppression of FLT3-dependent survival were demonstrated with lestaurtinib (97). These pre-clinical data were the supporting evidence to investigate lestaurtinib in clinical trials.

Similar to the lestaurtinib story, others investigated PKC412, a small-molecule developed as a protein kinase C inhibitor, but was found to have inhibitory activity against multiple class III RTK, including PDGFR, VEGFR, and FLT3 (106). PKC412 (midostaurin) inhibits the proliferation of transformed cell lines transfected with activating mutations of FLT3, and shows clinical activity in mice with FLT3-induced MPD (75, 106).

#### Sequential delivery of FLT3 inhibitors and chemotherapy

Incorporation of FLT3 inhibitors into existing chemotherapy regimens was studied pre-clinically, first in FLT3-ITD-expressing cell lines and primary patient AML blasts. *In vitro*, pre-treatment with lestaurtinib followed by chemotherapy agents is antagonistic, whereas simultaneous therapy or treatment with lestaurtinib after chemotherapy has a synergistic cytotoxic effect (107). FLT3 inhibitors arrest FLT3-activated cells in G1 phase of cell cycle, rendering these cells insensitive to drugs such as cytarabine and etoposide that exert their cytotoxic effects on cells in active S phase. Surprisingly, AML cells with either ITD or wild-type FLT3 showed synergistic cytotoxicity with lestaurtinib and daunorubicin. This effect is likely due to the competitive binding of these drugs to the same plasma protein, increasing their free concentrations, which could potentially increase systemic toxicity of daunorubicin. Treating cells with chemotherapy first, followed by lestaurtinib, surmounts both of these undesirable combination effects. Similar combination experiments in infant MLL-r ALL cells overexpressing wild-type FLT3 were performed, and again synergy was observed using chemotherapy first, followed by lestaurtinib (108). These models provided invaluable guidance moving forward with FLT3 inhibitors in clinical trials.

#### Development of correlative assay to determine efficacy (PIA)

The hypothesis of kinase inhibitors in malignancies is that inhibition of the kinase receptor signaling will result in anti-tumor effect and concomitant clinical response. However, acquiring tissue to compare signaling changes before and after therapy is challenging. Additionally, plasma drug concentration does not necessarily reflect therapeutic efficacy, since variances in plasma protein binding affects the level of free/active drug. For these reasons, a useful surrogate *in vitro* assay was developed to assess the efficacy of FLT3 inhibition in patients. A plasma inhibitory assay (PIA) utilizes an FLT3-ITD transfected cell line, TF1-ITD, mixed with patient plasma at various time points during treatment, to measure levels of FLT3 phosphorylation (109). This method reliably correlated with patient responses to treatment. In a phase 1 clinical trial with lestaurtinib, a PIA of 85% or greater correlated with patient clinical response to drug and reflects an approximate threshold for FLT3 inhibition *in vivo* (110).

### FLT3 inhibitors in leukemia

This section will summarize the best-characterized FLT3 inhibitors in use in pediatric leukemia, and discuss some relevant adult clinical trial data. A summary of recently completed or ongoing pediatric clinical trials with FLT3 inhibitors, along with the NCT identifying numbers, is shown in Table 1.

**Table 1:** **Pediatric clinical trials with FLT3 inhibitors: recently completed or ongoing studies**.

Trial identifier	Phase	Status	Patients	Drug(s)	Results
NCT00469859 COG AAML06P1	1/2	Completed	Relapsed AML	Lestaurtinib (CEP-701) Cytarabine Idarubicin	Not published
NCT00557193 COG-AALL0631	3	Completed	Newly diagnosed ALL Infants <12 m	Lestaurtinib (CEP-701) Chemotherapy (modified P9407)	Not published
NCT00866281	1/2	Recruiting	MLL-r infant ALL FLT3-mutant AML	Midostaurin (PKC412)	N/A
NCT01411267 TACL 2009-004	1	Completed	Relapsed/refractory ALL or AML	Quizartinib (AC220) Cytarabine Etoposide Methotrexate	Not published; Abstract presented at ASH 2013
NCT00908167 St. Jude RELHEM	1	Recruiting	Relapsed/refractory ALL or AML	Sorafenib Cytarabine Clofarabine	N/A; although pilot cohort data published: Inaba et al. (111)
NCT00665990 St. Jude ANGIO1	1	Recruiting	Refractory ALL, AML or solid tumors (solid tumor portion completed)	Sorafenib Bevacizumab Low dose-cytoxan	N/A
NCT01371981 COG AAML1031	3	Recruiting	Newly diagnosed AML	Sorafenib Bortezomib Chemotherapy (ADE or ADE/MA based)	N/A
NCT01445080	1	Completed	Refractory ALL, AML or solid tumors	Sorafenib	Published; Widemann et al. (112)

*Trial information from www.clinicaltrials.gov referenced July, 2014*.

#### CEP-701/lestaurtinib

##### CEP-701/lestaurtinib in adult leukemia

The first use of FLT3 inhibitors in leukemia patients was a small phase 1/2 trial of lestaurtinib monotherapy in adult patients with heavily pretreated, refractory/relapsed AML. Five of 14 patients achieved some clinical response, with a significant decrease in marrow or circulating peripheral blasts, and little observed toxicity. PIAs were performed on patient plasma as described above, and demonstrated inhibition of FLT3 activity down to a target level of 10–15% in 8 of 14 evaluable patients; 5 of these were the patients with clinical responses. Interestingly, the other patient samples with good FLT3 inhibition did not demonstrate cytotoxicity *in vitro* or clinical response, suggesting a resistant phenotype. Clinical responses lasted 2 weeks to 3 months (110).

A phase 2 study of lestaurtinib followed, in older adults with newly diagnosed AML who were unsuitable candidates for conventional chemotherapy (113). Drug was again generally well tolerated with mild GI toxicities. Two of 29 patients had grade 4 thrombocytopenia, and one died from a CNS hemorrhage. Three of 5 patients with mutant FLT3 and 5 of 22 patients with wild-type FLT3 experienced some clinical response with decreased blasts, which again correlated with the degree of FLT3 inhibition by PIA data. Again, responses with monotherapy were short-lived, generally only sustained for several weeks.

A randomized phase 2 study of salvage chemotherapy in combination with lestaurtinib was completed in adult patients with FLT3-mutant AML in first relapse (114). There was no difference in OS between the two arms, but importantly, only 58% of patients treated with lestaurtinib achieved FLT3 inhibition down to target levels, and only 27% sustained FLT3 inhibition. FLT3 inhibition correlated well with drug response, whereas plasma drug concentration did not correlate well with response. These results validate the importance of the correlative laboratory studies in determining patient response to FLT3 inhibitors.

##### CEP-701/lestaurtinib in pediatric leukemia

Based on the pre-clinical data in pediatric leukemia with either activating FLT3 mutations or overexpression, lestaurtinib was moved into pediatric trials. In 2007, the COG opened a phase 1/2 pilot study of lestaurtinib in relapsed/refractory AML patients with FLT3 mutations. Dose level 1 was 50 mg/m2 and dose level 2 was 62.5 mg/m2, given orally, in combination with idarubicin and cytarabine. Based on the drug-sequence pre-clinical data, chemotherapy was given on days 1–4, and lestaurtinib given on days 5–28. The study completed accrual in 2010, and data are not yet published. Primary outcomes studied were safety/tolerability and >80% inhibition of FLT3 phosphorylation in the majority of patients at trough time points.

A phase 3, randomized COG trial with lestaurtinib, in combination with standard chemotherapy, opened in 2008 for newly diagnosed infant ALL (COG-AALL0631). The study has recently completed, and results are pending. Intermediate risk (MLL-r and >90 days old) and high-risk patients (MLL-r and <90 days old) were randomized to receive lestaurtinib after induction therapy, with a primary study outcome of EFS. Secondary outcomes include correlative laboratory studies, such as PIAs to determine inhibition of FLT3 phosphorylation. MLL wild-type infants did not receive lestaurtinib.

#### PKC412/midostaurin

##### PKC412/midostaurin in adult leukemia

A phase 2 clinical trial of midostaurin in 20 adults with relapsed or refractory FLT3-mutated MDS or AML showed that drug was generally well tolerated, though 2 patients suffered fatal pulmonary events of uncertain etiology (115). Peripheral blood blasts decreased by at least 50% in 14 (70%) of patients, and this result was sustained for at least 4 weeks in 7 (35%) patients. PIAs were not used in this trial, but peripheral blasts recovered at early time points demonstrated inhibition of FLT3 phosphorylation in patients with good clinical responses.

This was followed by a phase 2B study in 95 adults with relapsed or refractory AML or those unable to receive conventional chemotherapy. Patients had wild-type or mutated FLT3 and were randomly assigned to receive oral midostaurin monotherapy at either 50 or 100 mg twice daily. Interestingly, 71% of patients with mutant FLT3 experienced a hematological response with at least 50% reduction in peripheral blasts, whereas only 42% of patients with wild-type FLT3 demonstrated a clinical response (116). A phase 1B study combined midostaurin with standard induction chemotherapy in three different schedules, in patients with newly diagnosed AML. At tolerable doses of 50 mg twice daily, there was an 80% complete response (CR) rate in these patients; specifically, 74% CR rate in FLT3-wild-type patients and 92% CR rate in FLT3-mutant patients (117). These results provided rationale for a large, recently completed phase 3 clinical trial of midostaurin for adult AML with FLT3-mutant leukemia (clinical trial identifier NCT00651261); results are not yet available^1^.

##### PKC412/midostaurin in pediatric leukemia

In pediatrics, a phase 1/2 clinical trial open in Europe and some U.S. centers is currently recruiting MLL-r infant ALL and FLT3-mutant AML patients to receive midostaurin as a single agent, with a primary outcome of determining the maximally tolerated dose (MTD). Secondary outcomes include safety/tolerability, PKs, preliminary efficacy of the drug in these diseases, and laboratory correlatives, including evaluation of FLT3 phosphorylation before and after drug.

#### AC220/quizartinib

Quizartinib, a small-molecule inhibitor engineered specifically for FLT3, is incredibly potent and selective compared to the previous “first-generation” FLT3 inhibitors, with low nanomolar concentrations of quizartinib achieving inhibition *in vitro* (118, 119).

##### AC220/quizartinib in adult leukemia

In a phase 1 adult study, 76 patients with relapsed or refractory AML received quizartinib monotherapy (120). Hematological responses were seen in 53% of patients with FLT3-ITD mutations, and PIAs revealed near-total inhibition of FLT3 phosphorylation in nearly all cases. Subsequently, a phase 2 study of relapsed/refractory AML patients, all containing ITD mutations, recently completed accrual, and results were presented in abstract form at the 2012 American Society of Hematology (ASH) meeting (121, 122). As monotherapy, quizartinib impressively induced a CR or a CR with incomplete blood count recovery (CRi) in 9 of 17 patients (53%). Ongoing adult studies are now evaluating quizartinib in combination with chemotherapy (NCT01390337)^1^.

##### AC220/quizartinib in pediatric leukemia

The Therapeutic Advances in Childhood Leukemia and Lymphoma (TACL) consortium recently completed a pilot study of quizartinib in combination with cytarabine and etoposide, in pediatric patients with MLL-r ALL or relapsed/refractory AML. Chemotherapy was administered on days 1–5 and oral quizartinib was given days 7–28, for up to two courses of therapy. PIAs were obtained to determine biological efficacy of FLT3 inhibition. Preliminary results were presented at the 2013 annual ASH conference and showed good tolerability of drug (123). The correlative PIA assays demonstrated near-total (>99%) inhibition of FLT3 phosphorylation after quizartinib. Importantly, four of six patients with FLT3-ITD-mutant AML achieved a CR or CRi and the other two ITD patients had stable disease. These results support further testing of quizartinib in pediatric leukemia, particularly in FLT3-ITD-mutant AML.

#### Sorafenib

##### Sorafenib in adult leukemia

Three phase 1 trials with sorafenib in adults with relapsed/refractory AML have been published to date. The first study treated 16 patients and found a hematological response (>50% reduction in peripheral or BM blasts) in patients with ITD mutations but not in those with wild-type FLT3 (124). The second study included one patient with an ITD mutation who achieved a CR with sorafenib alone (125). In the third study, the best response was stable disease in 11 of 15 patients, but 2 of these 11 were the only patients with ITD mutations (126).

A phase 1/2 adult study evaluated 10 relapsed AML patients with sorafenib in the dose-finding phase, and then treated 51 newly diagnosed AML patients with a combination of sorafenib, cytarabine, and idarubicin (127). PIAs demonstrated complete inhibition of FLT3 phosphorylation. Impressively, 38 (75%) of all patients achieved a CR, and all patients with ITD mutations achieved a CR or a CRi. One year-OS rate for all patients was 74%. A phase 2 German study randomized elderly AML patients to receive placebo or sorafenib, followed by cytarabine (128). No differences in EFS or OS were noted, but this study was limited by the patient population and the relatively small number of ITD-positive patients (28 of 197 or 14%).

##### Sorafenib in pediatric leukemia

Recent early phase pediatric clinical trial reports with sorafenib appeared promising. Inaba et al. published results from a pilot group of 12 patients with relapsed/refractory AML. Patients received sorafenib in combination with clofarabine and cytarabine in the following manner: oral sorafenib on days 1–7, sorafenib plus IV clofarabine/cytarabine on days 8–12, and single-agent sorafenib on days 13–28 if tolerated (111). Grade 3 dose limiting toxicity (DLT) of hand–foot skin reaction was reported at a dose of 200 mg/m2, thus 150 mg/m2 was subsequently used and well tolerated. Five of five patients with ITD mutations achieved a CR or CRi, and three of seven patients with wild-type FLT3 also achieved a CR. *In vitro* inhibition of FLT3 downstream signaling protein phosphorylation was demonstrated in most of these patient samples upon treatment with sorafenib. Interestingly, conversion of sorafenib to its active metabolite, sorafenib N-oxide, was three to four times higher in children than previously reported in healthy adult and adults with leukemia. Although this may have contributed to increased non-DLT skin reactions seen in children, the active metabolite is also a more potent inhibitor of FLT3-ITD (111).

Watt and Cooper described three case reports of pediatric relapsed/refractory AML that achieved sustained remissions with sorafenib in combination with chemotherapy. Significant toxicities were noted in two of three patients, but efficacy was demonstrated in each case. Sorafenib combination therapy served as a bridge to transplant in one of these cases, a 7-year old male with ITD-mutant AML, and that patient remains in remission 21 months post-transplant (129). Finally, a COG phase 1 consortium trial at the NCI investigated single-agent sorafenib in refractory solid tumors or leukemia. The MTD for solid tumors and leukemia are 150 and 200 mg/m2 twice daily, respectively. Eleven leukemia patients (eight AML and three ALL) were enrolled. Two AML patients with ITD mutations achieved a CR and went off study to undergo stem cell transplantation (112).

Given these encouraging results, the current phase 3 COG randomized trial for newly diagnosed AML incorporates sorafenib for pediatric FLT3-ITD AML. This trial non-randomly assigns ITD-mutant patients with high allelic ratios (>0.4, as previously discussed, HR-ITD) to Arm C, which includes sorafenib in addition to chemotherapy. Simultaneous sorafenib begins immediately in induction therapy and continues throughout the treatment protocol. It is recommended for all high-risk patients to receive a stem cell transplant in first CR, if any suitable donor is available. For HR-ITD patients, this trial also includes a maintenance phase with single-agent sorafenib for 1 year, beginning between day 40–80 either after transplant or after finishing chemotherapy.

#### ASP2215

ASP2215, a promising new small-molecule inhibitor, demonstrated potent kinase inhibition of FLT3, LTK, ALK, and AXL (130). Furthermore, ASP2215 inhibited the growth of cell lines expressing FLT3-ITD or FLT3-D835-TKD mutations. The same group also showed that ASP2215 induced complete tumor regression in MV4;11 xenografts as a single agent (130) and in combination with chemotherapy (131). ASP2215 is currently being investigated in adult relapsed/refractory AML with a multi-institutional phase 1/2 trial in the United States (NCT02014558) and a phase 1 trial in Japan (NCT02181660)^1^.

### Other therapeutic approaches in FLT3-activated leukemia

#### Role of HSCT in first remission in patients with FLT3-ITD mutations

The beneficiary role of HSCT in first remission for FLT3-ITD AML has been controversial, although due to generally poor outcomes in this cohort with chemotherapy alone, HSCT is rapidly becoming the standard of care (132, 133). With improved transplant-related mortality in recent years, the benefit of HSCT in pediatric high-risk AML has become more apparent. Two recent pediatric reports show no significant difference in outcomes between high-risk (including FLT3-ITD patients) and standard-risk AML patients receiving HSCT in first CR, demonstrating that HSCT abrogates the poor outcomes associated with high-risk pediatric AML (134, 135). The current phase 3 COG randomized trial for newly diagnosed AML, as mentioned, non-randomly assigns HR-ITD patients to Arm C with sorafenib. Patients may continue on study as long as they achieve and maintain remission status (<5% blasts at end of induction II or better). After intensification I, these patients may proceed with allogeneic HSCT if any appropriate donor is available, and if no donor is available, they may receive another intensification cycle (with ongoing sorafenib). This is not a randomized design, but the outcomes of these patients compared to historical controls are hypothesized to show improvement with HSCT.

#### Role for maintenance FLT3 inhibitor after hematopoietic stem cell transplantation

##### Adult leukemia

There are good data in the adult literature to suggest a role for sorafenib as maintenance therapy in FLT3-ITD AML, particularly after HSCT. Several smaller studies of relapsed adult FLT3-ITD AML post-HSCT reported some CRs and other good responses with single-agent sorafenib after allogeneic HSCT (136–138). A recent retrospective review of 65 FLT3-ITD AML patients compared single-agent sorafenib efficacy in newly relapsed patients who had previously received conventional chemotherapy versus those who had a prior HSCT (139). In this series, the patients who had received a prior HSCT achieved longer remission before treatment failure compared to those receiving chemotherapy previously (197 versus 136 days, *p* = 0.0305) and also achieved “deeper” remissions, with a strikingly 24% of post-HSCT patient achieving a complete molecular remission (compared to 8% with prior chemotherapy). The authors of this series and others have proposed a possible immuno-modulatory effect of sorafenib after transplant in these patients, though a mechanism for this effect is not yet elucidated. Ongoing adult clinical trials are studying post-transplant sorafenib in greater detail (NCT01578109, NCT01398501). Also, a phase 1 study is evaluating quizartinib as maintenance therapy after allogeneic transplant in adult AML (NCT01468467), and a phase 2 study is investigating midostaurin as maintenance after allogeneic transplant in FLT3-ITD adult AML (NCT01883362)^1^.

##### Pediatric leukemia

There is limited but promising experience with post-HSCT sorafenib in pediatric AML. In Seattle, 15 FLT3-ITD pediatric AML patients received sorafenib post-HSCT: 10 after relapse and 5 prophylactically. Seven of the 10 relapsed patients remain in a second CR at a median of 12.5 months post start of therapy, and 4 of the 5 patients given sorafenib prophylactically remain disease-free at a median of 10 months (Pollard, unpublished data). Interestingly, one patient who received prophylactic sorafenib post-HSCT was discovered to have an ITD-negative clone at relapse, suggesting clonal evolution. The current phase 3 COG-AAML1031 trial incorporates maintenance FLT3 inhibitor therapy after transplant. FLT3-ITD patients on this study will have received sorafenib in pre-HSCT therapy, and upon count recovery post-HSCT, will resume sorafenib as a single agent until day 364 post-transplant. Effectiveness of this novel maintenance FLT3 inhibitor therapy is yet to be determined. Also, the effect of sorafenib on graft versus host disease, and therefore, potentially graft versus leukemia effect, is not known. The role of other FLT3 inhibitors, such as quizartinib, after stem cell transplant in FLT3-activating leukemia has not yet been addressed in the pediatric population.

## Challenges and Future Approach to FLT3 Inhibition

### FLT3 inhibitor resistance: Both challenge and validation of FLT3 addiction

Despite promising biological activity in pre-clinical models and good clinical activity of various FLT3 inhibitors in early phase clinical trials, effective therapeutic targeting of FLT3 has proved challenging. Resistance to targeted therapy often develops due to pharmacokinetic (extrinsic) or pharmacodynamic (intrinsic) forces. The mechanisms of resistance to FLT3 inhibitors can be further classified as extrinsic, receptor-intrinsic, or cell-intrinsic (140). Primary resistance occurs from the onset of treatment, and secondary resistance occurs at relapse after initial response to FLT3 inhibitors.

#### Extrinsic mechanisms: pharmacokinetic barriers

Trials with first-generation FLT3 inhibitors showed difficulty achieving adequate plasma free drug concentrations. Hepatic CYP3A4-mediated metabolism of midostaurin results in two metabolites with decreased FLT3-specific activity. Midostaurin and its metabolites can also induce CYP3A4, resulting in highly unreliable concentrations of drug (141). Midostaurin must therefore be given with caution with other medications affecting CYP3A4 activity. Lestaurtinib is particularly highly bound to plasma protein, and competes with anthracyclines for alpha-1 acid glycoprotein binding sites, resulting in decreased free concentration of lestaurtinib, and potentially increased anthracycline toxicity. Also, medications such as azole antifungals can affect the metabolism of certain FLT3 inhibitors (142).

#### Cell-intrinsic mechanisms

##### Activation of alternate pathways and survival signaling

Primary resistance to FLT3 inhibitors can occur through the activation and up-regulation of alternate survival signaling pathways. Anti-apoptotic pathways involving Bcl-x and BCL2 are differentially activated in FLT3-ITD compared to FLT3-wild-type AML (143). Interestingly, BCL2 is not down-regulated in FLT3-ITD cells upon FLT3 inhibitor treatment, signifying a mechanism of primary resistance. Treatment with the BH3 mimetic ABT737 restored sensitivity to FLT3 inhibition, suggesting a role for dual targeting of FLT3 and BCL2 (144). MV4;11 cells are homozygous for ITD, and upon developing resistance to midostaurin, demonstrate up-regulation of anti-apoptotic genes MCL-1 and C-KIT by microarray and PCR compared to parental MV4;11 cells (145). Knapper et al. demonstrated that AML primary cells show variable cytotoxicity to FLT3 inhibition with midostaurin or lestaurtinib and retain activation of STAT5 and MAPK pathways even with near-complete inhibition of FLT3 phosphorylation (146). In another investigation, human AML cell lines in prolonged culture with FLT3 inhibitors were selected for resistant clones, and sequencing of these clones revealed no secondary TKD mutations. FLT3 was completely inhibited in these resistant cells, yet the phosphorylation and activation of STAT5, AKT, and/or MAPK was maintained (147). Furthermore, MEK or PI3K inhibition restored sensitivity of these cells to FLT3 inhibition. Piloto et al. also found that two of the resistant cell lines had developed new, activating N-Ras mutations that were not present in the parental sensitive cell line. Taken together, these reports are supporting *in vitro* evidence for the activation of FLT3-independent pathways, promoting cell survival and proliferation during FLT3 inhibitor therapy.

Another proposed mechanism of resistance involves the FL. Sato et al. showed that FL levels are significantly higher following intensive chemotherapy, and are strikingly high in relapsed AML patients (148). Adding exogenous FL to AML samples *in vitro* (at levels similar to that seen in patients) conferred relative resistance to five different FLT3 inhibitors. This spike of FL after chemotherapy may therefore represent an obstacle to FLT3 inhibition, and suggests a role for the development of novel FL inhibitors. Reducing chemotherapy intensity could attenuate the rise in FL and may be an important consideration. In another model, investigators compared the gene-expression profiles of MV4;11 cells resistant or sensitive to FLT3 inhibitor ABT869 (149). The resistant cells showed up-regulation of FL and survivin, and up-regulated survivin led to decreased apoptosis and increased number of cells in S phase. They propose that an increase in FL in the resistant cells caused increased STAT activation, which in turn led to increased survivin. This group further showed that survivin expression was directly regulated by activated STAT3. Combining ABT869 with the STAT inhibitor IDRE804 reversed this effect, and the combination demonstrated therapeutic synergy *in vivo*.

##### Microenvironment and stromal protection

Clinical studies with single-agent FLT3 inhibitors demonstrate good clearance of circulating peripheral blasts, but a marginal or delayed effect on blasts in the BM, suggesting a protective role of the BM microenvironment. Leukemic stem cells (LSC), like normal hematopoietic stem cells, reside in stromal “niches” with specialized conditions for optimal growth and survival (150). Sequestered in these niches, it is hypothesized that LSCs may evade chemotherapy or targeted therapy-induced cell death. Interestingly, in stroma-like conditions *in vitro*, FLT3-ITD AML blasts become resistant to FLT3 inhibitor effects, and in fact demonstrate expansion (151, 152). CXCR4 and its ligand, SDF-1, are important regulators of stromal/leukemia cell interactions (153, 154). FLT3 and its ligand FL have been shown to modulate surface CXCR4 expression, which is up-regulated in FLT3-ITD AML patient samples (155, 156). Subsequently, pre-clinical models have shown that CXCR4 inhibition enhances the sensitivity of FLT3-ITD AML cells to FLT3 inhibition (157, 158). An ongoing phase 1 trial in adults is investigating the combination of CXCR4 inhibitor AMD3100 (Plerixafor) and sorafenib in relapsed/refractory FLT3-ITD AML (NCT00943943). It has been demonstrated that Plerixafor enhances the response of MLL-r ALL cells to FLT3 inhibition in xenografts, suggesting that this dual targeting may prove useful in this high-risk subset of pediatric leukemia patients (159).

#### Receptor-intrinsic mechanisms

##### Acquired point mutations

In BCR-ABL leukemia, the selection of clones that harbor mutations in the ATP-binding pocket and prevent the binding of imatinib is the most common cause of drug resistance (160). Likewise, the development of TKD point mutations at relapse after FLT3 inhibitor therapy is well described, and is a significant mechanism of secondary resistance in FLT3-ITD AML patients (161). This phenomenon is compelling evidence that FLT3-ITD represents a driver mutation in AML, and that the ITD activating mutation can render a state of “oncogene addiction” in this disease. Prior to reports of acquired mutations in patient samples, forced resistance to FLT3 inhibitors *in vitro* predicted the development of acquired TKD mutations at certain key residues (162, 163). Many of these have since been confirmed in patients, and Table 2 summarizes acquired point mutations that have been described after FLT3 inhibitor therapy. The first case report described a novel FLT3-TKD mutation N676K, discovered at relapse in an FLT3-ITD AML patient after midostaurin monotherapy (164). 32D cells transfected with ITD-N676K FLT3 confirmed *in vitro* resistance to midostaurin, relative to cells transfected with FLT3-ITD. The second report described a FLT3-ITD-positive, BCR-ABL-negative CML patient in blast crisis, who acquired the mutation A848P at relapse after a 9-month response to alternating therapy with sunitinib and sorafenib (165). Ba/F3 cells with ITD-A848P FLT3 were resistant to both sunitinib and sorafenib, but maintained sensitivity to midostaurin. Neither mutation was detected prior to FLT3 inhibitor therapy. Additional acquired mutations are likely to emerge, based on screening *in vitro* models, such as the highly resistant Y842C mutation (166).

**Table 2:** **Acquired point mutations in FLT3-ITD patients after FLT3 inhibitor therapy**.

Mutation	Disease	Therapy	Reference	Validated *in vitro*
N676K	AML	PKC412	Heidel et al. (164)	32D
A848P	CMML Relapsed blast crisis	Sunitinib and sorafenib	von Bubnoff et al. (165)	Ba/F3
F691L	AML	AC220	Smith et al. (161)	Ba/F3
	AML	AC220	Albers et al. (167)	Ba/F3
	AML (pediatric)	Sorafenib	Baker et al. (168)
D835Y	AML	AC220	Smith et al. (161)	Ba/F3
	AML	Sorafenib	Man et al. (169)
	AML (pediatric)	Sorafenib	Baker et al. (168)
D835V	AML	AC220	Smith et al. (161)	
D835F	AML	AC220	Smith et al. (161)	
D835H	AML	Sorafenib	Man et al. (169)	Ba/F3
	AML (pediatric)	Sorafenib	Baker et al. (168)
D651G	AML	Sorafenib	Zhang et al. (170)	
G619C	AML	Sorafenib	Zhang et al. (170)	
I687F	AML	Sorafenib	Zhang et al. (170)	
E858K	AML	Sorafenib	Zhang et al.(170)	

A recent report evaluated paired pre-treatment and relapsed samples from eight adult FLT3-ITD AML patients treated with quizartinib monotherapy, all of which relapsed after initial good marrow responses (161). Using single molecule real-time (SMRT) sequencing, the ITD alleles were analyzed and all eight patients had developed additional point mutations in the kinase domain at relapse that were not detected pre-treatment. Four patients had developed polyclonal resistance with multiple point mutations, validating the significant selective pressure of quizartinib on these leukemic clones. In this report, TKD mutations in patients were confined to residues F691 and D835. *In vitro* cell-based assays containing these mutations confirmed resistance to quizartinib, and demonstrated cross-resistance to sorafenib (161). Other reports have identified or confirmed TKD mutations after FLT3 inhibitor therapy with quizartinib (167) or sorafenib (168–170). Baker et al. described three pediatric AML patients who partially responded to sunitinib treatment after developing sorafenib-mediated TKD mutations D835H, D835Y, or F691L. However, two of these three patients had expansion of the D835Y population while on sunitinib, and this mutation was polyclonal, present on both ITD-positive and ITD-negative alleles (168). Interestingly, although many studies report that TKD mutations are not detected prior to FLT3 inhibitor therapy, emerging evidence suggests these TKD mutations may be present in a tiny sub-clone at pre-treatment, which is not easily detectable, but is selected for over time with FLT3 inhibitor therapy. Man et al. expanded LIC from pre-treatment patient samples in NOD/SCID mice. In these xenografts, D835Y-positive clones were expanded, which had only been detected in the paired post-sorafenib patient samples at relapse (169).

The patterns of TKD mutations and drug resistance are non-overlapping, suggesting that combinations of FLT3 inhibitors may partially overcome the development of resistance (171). Type I FLT3 inhibitors, lestaurtinib and midostaurin, bind to the active formation of the FLT3 receptor, whereas type II inhibitors bind to the inactive formation (172). Also, not all acquired TKD mutations confer resistance in the same manner. Ba/F3 cells with D835H/Y demonstrate IL-3-independent growth and increased STAT5 phosphorylation, consistent with reports that D835 mutations are primary transforming events (173). Ba/F3 cells with FLT3-F691L are not transformed, suggesting that F691 mutations are secondary events and confer resistance by interfering with drug binding. Most FLT3 inhibitors make direct contact with residue F691 (166); although sunitinib does not, which could explain its conserved sensitivity in F691 mutants (168). In contrast, much evidence suggests that type II inhibitors quizartinib and sorafenib are inactive against D835 mutations, likely due to the mutant’s stabilization of the FLT3 receptor in the active formation. Baker et al. showed that cells with FLT3-ITD-D835Y mutant were partially sensitive to midostaurin, which has type I inhibitor activity. However, the relatively lower potency of the first-generation type I inhibitors limit their efficacy as single agents. The next-generation FLT3 inhibitor crenolanib has shown promising efficacy against FLT3-ITD-D835Y AML samples, and is currently in a phase 2 clinical trial for adult AML (174). Taken together, these data argue for personalized selection and careful consideration of FLT3 inhibitor therapy in FLT3-ITD patients, and close monitoring for the emergence of TKD-mutant clones, which may be more susceptible to different FLT3 inhibitors.

##### Up-regulation of FLT3 receptor

Studies have shown increased expression of FLT3 itself in AML blasts during treatment with midostaurin (106) or lestaurtinib (113). It is not definitively known whether this observation represents a feedback loop that can act as a mechanism of resistance.

### Clonal evolution of the leukemic stem cell

Increasing evidence suggests that small populations of cancer stem cells can evade anti-neoplastic induced cytotoxicity (175, 176). Likewise in AML, genome-wide sequencing of paired diagnostic-relapsed samples reveal that multiple sub-clones are usually present at diagnosis, which have evolved from a “founding clone,” synonymous with the LSC. At relapse, a dominant sub-clone emerges, either because the founding clone has gained additional mutations or a particular sub-clone has survived initial therapy (177). The latter mechanism of relapse is likely to be shaped by whatever remission-inducing therapy the patient has received. *In vitro* comparison of FLT3 inhibitor cytotoxicity in diagnostic and relapsed FLT3-ITD patient samples demonstrate that FLT3 inhibitor therapy is relatively less cytotoxic at diagnosis, despite adequate inhibition of FLT3 phosphorylation. This suggests that at diagnosis in FLT3-ITD AML, the dominant clone may not always be reliant on FLT3 signaling. In contrast, relapsed AML cells with a higher ITD allelic burden are more sensitive to FLT3 inhibitors, suggesting that an ITD dominant clone is more likely to emerge at relapse (178).

In order for a targeted therapy to eradicate disease, the target itself must be present in the cancer stem cell, and it remains controversial whether FLT3-ITD mutations are present in the LSC. FLT3-ITD mutations are present in CD34^+^38^−^ leukemic initiating cells (38) and confer an engraftment advantage over non-FLT3-ITD AML in a xenograft model (179), suggesting that ITD mutations are present in the LSC. However, ITD mutations occur as both early and late “hits” in patients. In some cases, ITD mutations were present at diagnosis and lost at relapse, and in other patients, ITD mutations were acquired at relapse, providing evidence the ITD mutation occurred in a sub-clone and not the LSC (180, 181). Determining at which stage the ITD mutation arises has significant clinical implications regarding the strategic use and timing of FLT3 inhibitor therapy in AML.

### Future approaches

Careful treatment strategies are needed to increase the efficacy of targeted FLT3 therapy and decrease the development of resistance. The current approach to increasing efficacy has been to develop novel FLT3 inhibitors with increased potency and higher selectivity. However, as seen with quizartinib, these newer agents may increase the development of intrinsic resistance. Combining FLT3 inhibitors with chemotherapy improves responses, but does not preclude the development of resistance. When acquired mutations develop in BCR-ABL positive disease, the approach has been to switch to a different targeted agent. This approach could be taken in FLT3-ITD AML and would entail close monitoring for the development of TKD mutations while on FLT3 inhibitor therapy. Another approach could involve combining multiple FLT3 inhibitors that bind to different conformations of the receptor, in an attempt to prevent resistance. As discussed, some reports suggest that FLT3 inhibitors may be more effective in relapsed disease or in disease with a higher ITD allelic burden, and it might be prudent to reserve targeted therapy for those scenarios. Also, when FLT3 inhibitors are used in combination with chemotherapy, it may be possible to decrease the intensity of conventional chemotherapy drugs, and this might have the added benefit of lessening the FL surge demonstrated during more intensive chemotherapy. The development of a novel FL-inhibiting therapy would likely be synergistic with FLT3 inhibition. Perhaps, the most promising approach would be the dual inhibition of FLT3 and other pro-survival pathways, such as PI3K/AKT/mTOR, JAK/STAT5, or RAS/Raf/MEK/ERK inhibitors, and clinical trials investigating novel dual inhibitors (NCT02055781) or combining kinase inhibitors (NCT00819546) are underway^1^. Finally, combining FLT3 and CXCR4 inhibitors could overcome stromal protection of FLT3-activated leukemia cells and could be additive to any of the approaches above.

## Summary

The identification of FLT3 aberrancies in high-risk subsets of leukemia patients was discovered two decades ago, and much has been learned about the biology, clinical implications, and targeting possibilities. It remains controversial whether FLT3-ITD is an initiating event in leukemogenesis, yet the striking development of intrinsic resistance with FLT3 inhibitor therapy supports its role as one of the most meaningful cooperating events in the development of human AML (177). Both adult and pediatric clinical trials continue to investigate different FLT3 inhibitors in combination with chemotherapy in patients with FLT3-mutant AML. Also, in MLL-r infant ALL, wild-type FLT3 overexpression portends an especially poor prognosis, and FLT3 inhibitor therapy in these patients is being investigated in a phase 3 clinical trial through the COG. In the meantime, resistance to FLT3-targeted therapy poses a significant challenge in the treatment of FLT3-activated leukemia patients.

## Conflict of Interest Statement

The authors declare that the research was conducted in the absence of any commercial or financial relationships that could be construed as a potential conflict of interest.

## Footnotes

^1^www.clinicaltrials.gov (accessed on July, 2014).
